# Effect of Lymph Node Number on Survival of Patients with Lymph Node-Negative Gastric Cancer according to the 7th Edition UICC TNM System

**DOI:** 10.1371/journal.pone.0038681

**Published:** 2012-06-19

**Authors:** Dazhi Xu, Ying Huang, Qirong Geng, Yuanxiang Guan, Yuanfang Li, Wei Wang, Shuqiang Yuan, Xiaowei Sun, Yingbo Chen, Wei Li, Zhiwei Zhou, Youqing Zhan

**Affiliations:** 1 State Key Laboratory of Oncology in South China, Guangzhou, China; 2 Department of Gastric and Pancreatic Surgery, Sun Yat-sen University Cancer Center, Guangzhou, China; 3 Department of Radiotherapy, Sun Yat-sen University Cancer Center, Guangzhou, China; 4 Department of Hematology, Sun Yat-sen University Cancer Center, Guangzhou, China; Vanderbilt University Medical Center, United States of America

## Abstract

**Background:**

For the patients with node-negative gastric cancer, the 7th edition classification does not define the minimum number of lymph nodes necessary. We aimed to explore the prognostic significance of examined lymph nodes and determine how many nodes must be examined.

**Methodology/Principal Findings:**

435 patients underwent D2 gastrectomy with node-negative gastric cancer between December 1992 and December 2006 were obtained. Patients were classified into 4 groups by the number of negative LNs examined during surgery (1-6LNs, 7-10 LNs, 11-15 LNs, and > = 16 LNs). Stratified and Cox regression analyses were used to evaluate the association between survival and the number of negative LNs. Survival was significantly better in the > = 16 LNs, compared with the 1-5 LNs, 6-10 LNs and 11-15 LNs group in T2-4 patients; Multivariate analysis demonstrated tumor size, depth of invasion, 7th UICC stage and the number of examined nodes are strongly independent predictors of survival.

**Conclusions:**

This study first demonstrates that patients with lymph node-negative gastric cancer underwent D2 dissection should have at least 16 LNs examined, especially in advanced gastric cancer. These results are a reasonable supplement to our previous tumor-ratio-metastasis staging system and a stratification criterion in clinical pratice.

## Introduction

Gastric cancer is the fourth most common cancer worldwide [1]. The prognosis of gastric cancer patients remains poor, with a 5-year overall survival of 25% or less, especially in the USA, Europe, and China [2], [3]. Lymph node metastasis is an important prognostic indicator for the patients with gastric cancer. It is widely accepted that a higher survival rate benefits from a standardized pattern of lymph node dissection [4], [5], [6]. In 2010, the International Union Against Cancer (UICC) and the American Joint Committee on Cancer (AJCC) proposed the seventh edition of the UICC TNM classification with a substantial change in the staging of gastric cancer. Currently, the new classification is used most widely for the staging of gastric cancer [7]. But it does not define the minimum number of lymph nodes (LNs) necessary, especially for the gastric cancer patients stage pN0.

Although approximately 15% of the patients with node-negative disease still go on to die of disease [8], few studies have assessed the optimal examined number of LNs in patients with lymph node-negative gastric cancers according to the 7th UICC TNM System. The aim of the current study was to evaluate the long-term effect of the number of examined lymph nodes (LNs) on the prognosis of patients. We further explore the optimal number of LNs for accurate staging in the patients with node-negative gastric cancer after D2 dissection.

## Materials and Methods

### Patient Characteristics

**Figure 1:**
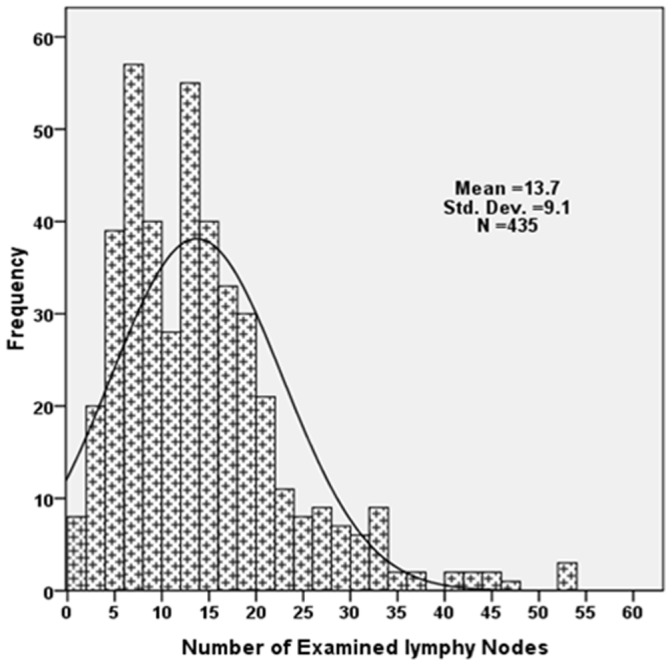
Distribution of the number of lymphy nodes examined for the entire cohort of 435 patients.

**Figure 2:**
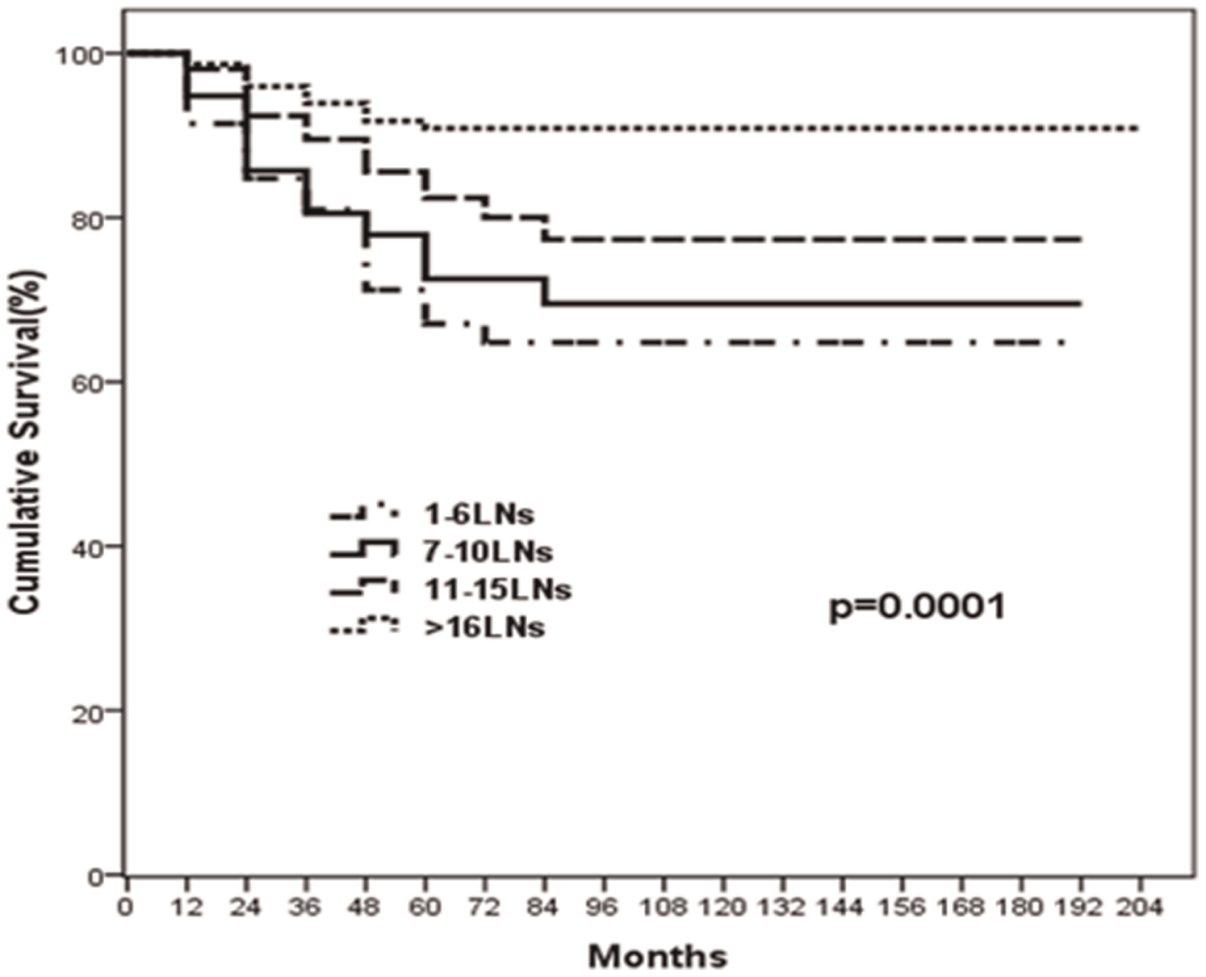
Overall survival curves for 4 categories of patients according to the number of the lymph nodes (LNs) examined in 435 patients (*P* = 0.0001).

A total of 1551 gastric carcinoma patients undergone D2 gastrectomy at Cancer Center of Sun Yat-sen University between December 1992 and December 2006 were selected. The eligibility criteria included histologically confirmed R0 resection, which was defined as no macroscopic and microscopic residual tumor and postoperative survival time ≥6 months. Patients who received chemotherapy or radiotherapy before surgery and patients with carcinoma of gastric stump were excluded from the study. D2 lymphadenectomy were performed by experienced surgeons following the Japanese Research Society for Gastric Cancer (JRSGC) guidelines [9]. At last, a total of 435 patients were enrolled in this study. We obtained informed consent from all participants involved in this study. Ethical approval was obtained from Sun Yat-sen University Cancer Center research ethics committee.

**Table 1:** Distribution of clinicopathologic characteristics for four categories by the number of examined lymph nodes.

Vatible	No. of examined nodes (n)				P
	1−6	7–10	11–15	> = 16	
Gender					0.191
Male	75(17.2%)	56(12.95)	72(16.6%)	90(20.7%)	
Female	30(6.9%)	21(4.8%)	33(7.6%)	58(13.3%)	
Age (years)					0.053
≤60	56(12.8%)	41(9.4%)	69(15.9%)	99(22.8%)	
>60	49(11.3%)	36(8.3%)	36(8.3%)	49(11.3%)	
Tumor size (cm)					0.167
≤5	66(15.2%)	50(11.5%)	66(15.2%)	106(24.4%)	
>5	39(9%)	27(6.2%)	39(9%)	42(9.7%)	
Tumor location					0.000
Lower	30(6.9%)	29(6.7%)	45(10.3%)	93(21.4%)	
Upper	67(15.4%)	38(8.7%)	41(9.4%)	34(7.8%)	
Middle	6(1.4%)	7(1.6%)	18(4.1%)	19(4.4%)	
Diffuse	2(0.5%)	3(0.7%)	1(0.2%)	2(0.5%)	
Depth of invasion					0.032
T1	17(3.9%)	12(2.8%)	24(5.5%)	44(10.1%)	
T2	19(4.4%)	17(3.9%)	17(3.9%)	26(6%)	
T3	18(4.1%)	15(3.4%)	24(5.5%)	38(8.7%)	
T4	51(11.7%)	33(7.6%)	40(9.2%)	40(9.2%)	
7th UICC stage					0.082
IA	17(3.9%)	12(2.8%)	24(5.5%0	44(10.1%)	
IB	19(4.4%)	17(3.9%)	17(3.9%)	26(6%)	
IIA	18(4.1%)	15(3.4%)	24(5.5%)	38(8.7%)	
IIB	48(11%)	31(7.1%)	38(8.7%)	36(8.3%)	
IIIB	3(0.7%)	2(0.5%)	2(0.5%)	4(0.9%)	

The median age of the cohort was 56 years (range, 16–83 years), and included 293 males and 142 females. All nodal material was separately dissected from the specimen at the end of the procedure by the surgeon. Each lymph node was submitted in separate pots labelled according to their site of origin, then measured in three dimensions and analyzed by the pathologist. For all the LNs, one section was routinely examined histopathologically. Sometimes serial sections were also cut from node area with the aim of the definited diagnosis and staging. Using the best cut-off approach in terms of the log-rank test, we classified the patients into four categories: 1-6 LNs, 7-10 LNs, 11-15 LNs and > = 16 LNs.

**Figure 3:**
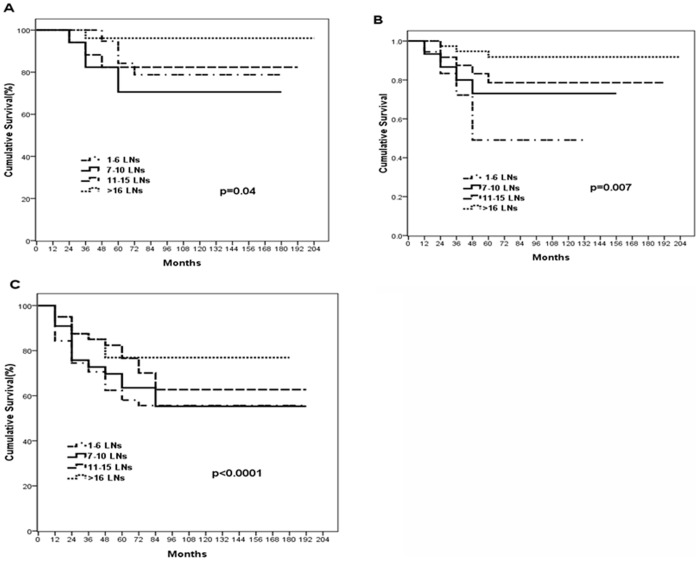
Gastric cancer-specific survival was found to be significantly higher among patients with >16 negative LNs compared with those with <16 LNs. (A) Gastric cancer-specific survival curves in pathological T2 patients according to the number of negative lymph nodes (LNs) (*P* = 0.040). (B) Gastric cancer-specific survival curves in pathological T3 patients according to the number of negative lymph nodes (LNs) (*P* = 0.007). (C) Gastric cancer-specific survival curves in pathological T4 patients according to the number of negative lymph nodes. (*P*<0.001).

### Follow-up

**Table 2:** Univariate analysis of variables in patients with node-negative gastric cancer.

Characteristics	5-year survival rate (%)	P
Gender		0.288
Male	76.5	
Female	82.4	
Age (years)		0.067
≤60	81.5	
>60	73.5	
Tumor size (cm)		0.000
≤5	89.2	
>5	57.1	
Tumor location		0.000
Lower	85.3	
Upper	73.9	
Middle	76	
Diffuse	25	
Depth of invasion		0.000
T1	97.9	
T2	83.5	
T3	77.9	
T4	64.6	
Number of examined LNs		0.000
1–6	65.7	
7–10	70.1	
11–15	79	
> = 16	91.2	
AJCC stage 7th		0.000
IA	97.9	
IB	83.5	
IIA	77.9	
IIB	66.7	
IIIB	36.4	

In general, all patients had follow-up after surgery every 3 months for the first year, every 6 months for the second year, and twice a year thereafter. The routine examination during follow-up included a physical examination, blood chemistry, X-ray of the chest, ultrasound of the liver and abdomen, bone scan and endoscopy. If the patient had specific symptoms, the examination was performed as soon as possible for a more careful assessment. The follow-up period ranged from the first day of therapy until death or until the last examination visit. The median follow-up period in our study was 72 months (range 6–197 months). We used disease-specific mortality for evaluating the association between the number of negative LNs and gastric cancer prognosis because it allows for controlling for unrelated causes of death [10]. The survival time was the time from diagnosis until the last contact, the date of death, or the date that the survival information was collected.

**Table 3:** Independent prognosic factor for cancer-specific survival by multivariate Cox regression analysis for the entire cohort of patients (n = 435).

Prognosicvariable	Relativerisk	95%CI for HR		P
		Lower	upper	
Tumor size (cm)				0.000
≤5	1			
>5	0.287	0.181	0.455	
Tumor location				0.931
Lower	1			
Upper	0.895	0.354	2.26	
Middle	0.952	0.344	2.639	
Diffuse	0.917	0.36	2.333	
Depth of invasion				0.000
T1	1			
T2	0.09	0.021	0.378	
T3	0.625	0.332	0.179	
T4	0.838	0.499	1.408	
Number of examined LNs				0.000
1–6	1			
7–10	4.262	2.182	8.322	
11–15	2.568	1.256	5.25	
> = 16	1.965	0.977	3.955	
AJCC stage 7th				0.000
IA	1			
IB	0.023	0.005	0.114	
IIA	0.193	0.075	0.508	
IIB	0.269	0.109	0.668	
IIIB	0.455	0.195	1.061	

### Statistical Methods

Survival analysis and curves were generated from observed postoperative survival times according to the Kaplan-Meier method and compared by the log-rank test. Multivariate analyses were carried out by the Cox proportional hazard model by the forward stepwise procedure for variable selection. Multivariate *P* values were used to characterize the independence of these factors. The 95% confidence interval (95% CI) was used to quantify the relationship between survival time and each independent factor. Differences were considered to be significant at the 5% level. All analyses were performed by SPSS for Windows, version 18.0 (SPSS, Chicago, IL). Spearman rank correlation coefficient was used to analyze the relationship between the number of examined LNs and recurrence rate. The correlation between the number of examined LNs and post-operative complication rate was analyzed with logistic regression model. Significance of differences was assumed at *P*<0.05.

## Results

### Patient Characteristics

A total of 435 cases fit the inclusion criteria and were included in the analysis. According to Martingale residuals of the Cox model, the cut-off points of the number of examined LNs was identified as 1–5, 6–10,11–15 and > = 16 LNs. Table 1 gives the characteristics of patients and their tumors according to the number of examined lymph nodes. Tumor location and depth of invasion had a significant inﬂuence on the number of examined lymph nodes. There were no significant differences between the distribution of gender, age, tumor size or 7th UICC stage and the 4 different categories according to the number of negative LNs.

### Number of Examined LNs and Survival

Fig. 1 shows the frequency distribution of examined LNs for the entire cohort of patients. The mean ± standard deviation number of pathologically examined LNs for the entire cohort of 435 patients was 13.5±4.5, ranging from 1 to 53 (median 12). As shown in Fig. 2, cancer-specific survival was significantly higher with an increasing number of negative LNs. The 5-year gastric cancer-specific survival rate was 65.7% for patients with 1 to 6 negative LNs compared with 70.1%, 79% and 91.2% for those with 7 to 10, 11 to 15 and more than16 LNs examined, respectively (*P*<.001).

### Identification of Optimal LN Number

Table 2 shows univariate survival analysis of variables in patients with node-negative gastric cancer. It revealed that tumor size, tumor location, depth of invasion, number of examined LNs, 7th UICC stage are associated with gastric cancer-specific survival.

Subgroup analysis was then conducted to evaluate the survival of the patients in different pathologic T categories. For patients with T2 tumors, the gastric cancer-specific survival difference was statistically significant between (<16 vs > = 16 negative LNs) (Fig. 3A). Simiarly, among T3 or T4 cases, patients with > = 16 negative LNs had the best disease-specific survival rates (Fig. 3B and 3C). The gastric cancer-specific survival in the patients with T1 tumors has no statistically difference. (data not shown).

### Survival Analysis

To identify whether the number of negative LNs was associated with survival, a multivariate logistic regression model was then applied (Table 3). The results demonstrated that: tumor size, depth of invasion, 7th UICC stage and the number of examined nodes is strong independent predictors of survival. Compared to the patients who had fewer than 6 examined nodes, patients with 6–10,11–15 and > = 16 LNs examined had a significantly lower chance of death during their gastrectomy.

## Discussion

Previously, we found that positive lymph node ratio (LNR) is an independent prognostic factor in gastric cancer after D2 resection regardless of the number of retrieved lymph nodes [11]. Furthermore, we demonstrated tumor-ratio-metastasis (TRM) staging system is an alternative to the 7th edition UICC TNM system in gastric cancer after D2 resection [12]. These results make it possible that a staging system based on the TRM could substitute for the current UICC classification, especially when the examined number of lymph nodes is not enough. However, it is difficult to evaluate the prognosis of patients by TRM for lymph node-negative gastric cancer [13].

In present study, all the node-negative gastric cancer patients underwent D2 resection, which is widely performed as a standard surgical procedure for gastric cancer in Asian countries. In order to assess the effect of the number of negative LNs on gastric cancer prognosis, disease-specific survival is used in our analyses to control for unrelated causes of death. The findings showed that the patients have a better disease-specific survival rate with increasing number of examined negative LNs. There were several potential reasons for that. First, some patients pathologically negative nodes in fact may have had cancer disseminated to regional LNs. Although all the patients underwent D2 dissection, the pathologists and surgeons vary in efforts and techniques in searching for lymph nodes, which could lead to omitted nodes in the specimen [14], [15]. As the number of LNs examined increases, the probability of missing a positive LN decreases and so does the proportion of patients with higher-stage disease who are misclassified as lower-stage. A low LNs examined results in an underestimation of stage, which is known as the Will Rogers phenomenon [10], [16]. Second, the contribution of negative node number to the prognosis of patients is partly due to considerably high rate LN micrometastases [17]. In node-negative patients identified by routine histologic examination, about 17%–32% had LN micrometastases [18], [19]. The patients with micrometastases often have an especially high risk for recurrence [20].

In current study, our data remarked depth of invasion and the number of examined nodes is strong independent predictors of survival in multivariate logistic regression model, which is similar to other studies [8], [20]. Moreover, the number of examined lymph nodes is associated with depth of invasion (T stage). Therefore, we analyzed the survival differences between groups according to different T stage. Compared to the patients who had fewer than 6 examined nodes, patients with 6–10,11–15 and > = 16 LNs removed had a significantly lower chance of death in patients with T2–4 disease. These patients are also defined as advanced gastric cancer, which constitutes the majority of gastric patients in clinical practice [21].

As for T1 stage gastric cancers in present study, the number of T1 cases is small and the 5-year survival rate is up to 97%, thus the gastric cancer-specific survival among them has no statistically difference. Additionally, the alternative explanation for this may be that patients with T1 classification seldom spread to regional LNs. The incidence of node metastasis in T1 patients about is only 15%–20% [22], [23]. However, it is difficult to determine depth of invasion and lymph node metastasis before surgery. Therefore, our data strongly suggest that at least 16 LNs should be removed during D2 resection.

Recently, several articles have evaluated prognostic value of lymph node number in lymph node-negative gastric cancer patients. Bouvier showed that at least 10 lymph nodes must be examined in order to accurately stage gastric carcinoma in node-negative cancers [24]. However, in their studies, most patients consisted underwent D1 resection, different from the current patients with only D2 resection. In the past, as some trials failed to demonstrate a survival benefit of D2 over D1 dissection, D1 lymphadendctomy resection was always performed in the Western countries [25], [26]. Whereas oriental surgeons do not accept the results and routinely perform D2 gastrectomy [27]. In fact, with the release of the latest clinical trial results, D2 lymphadenectomy is gradually becoming the recommended surgical approach for patients with resectable gastric cancer [28]. Baiocchi et al. also assessed 301 lymph node-negative gastric carcinoma patients with D2 resection [20]. They identified retrieval of more than 25 nodes may be warranted. Compared with our results, their selected were the patients with more than 15 nodes pathologically analyzed. However, many population-based studies of gastric cancer have found that surgeons and pathologists failed to accomplish more than 15 nodes [29], [30], [31]. Therefore, it is very important and meaningful to evaluate prognostic value less than 15 nodes of in lymph node-negative gastric cancer.

In addition, none of the above studies described the method of selecting of the reported cut-offs among the above studies. In this study, we examined the functional form of the covariate under study by Martingale residual analysis and identified the cutoff points of the number of examined LNs as 1-6 LNs, 7-10 LNs, 11-15 LNs, and > = 16 LNs [32], [33]. We believe the method can discriminate the survival differences between groups and make our study objectively valid [34], [35], [36].

In conclusion, the current study first demonstrates that patients with lymph node-negative gastric cancer following D2 dissection should have at least 16 LNs examined, especially in advanced gastric cancer. It should be used as a reasonable supplement to our TRM staging system and a stratification criterion in clinical pratice according to the 7th Edition UICC TNM System.
